# Elevated Serum Fibroblast Growth Factor 21 in Humans with Acute Pancreatitis

**DOI:** 10.1371/journal.pone.0164351

**Published:** 2016-11-10

**Authors:** Vivek K. Shenoy, Kristin M. Beaver, ffolliott M. Fisher, Garima Singhal, Jody R. Dushay, Eleftheria Maratos-Flier, Sarah N. Flier

**Affiliations:** Department of Medicine, Beth Israel Deaconess Medical Center, Harvard Medical School, Boston, Massachusetts, United States of America; Universitat de Valencia, SPAIN

## Abstract

**Background:**

The metabolic regulator Fibroblast Growth Factor 21 (FGF21) is highly expressed in the acinar pancreas, but its role in pancreatic function is obscure. It appears to play a protective role in acute experimental pancreatitis in mice. The aim of this study was to define an association between FGF21 and the course and resolution of acute pancreatitis in humans.

**Methods and Principal Findings:**

Twenty five subjects with acute pancreatitis admitted from May to September 2012 to the Beth Israel Deaconess Medical Center (BIDMC) were analyzed. Serial serum samples were collected throughout hospitalization and analyzed for FGF21 levels by ELISA. Twenty healthy subjects sampled three times over a four week period were used as controls. We found that, in patients with pancreatitis, serum FGF21 rises significantly and peaks four to six days after the maximum lipase level, before slowly declining. Maximum FGF21 levels were significantly greater than baseline levels for acute pancreatitis subjects (1733 vs. 638 pg/mL, P = 0.003). This maximum value was significantly greater than the highest value observed for our control subjects (1733 vs. 322 pg/mL, P = 0.0002). The ratio of active to total FGF21 did not change during the course of the disease (42.5% vs. 44.4%, P = 0.58). Fold increases in FGF21 were significantly greater in acute pancreatitis subjects than the fold difference seen in healthy subjects (4.7 vs. 2.0, P = 0.01). Higher fold changes were also seen in severe compared to mild pancreatitis (18.2 vs. 4.4, P = 0.01). The timing of maximum FGF21 levels correlated with day of successful return to oral intake (R^2^ = 0.21, P = 0.04).

**Conclusions:**

Our results demonstrate that serum FGF21 rises significantly in humans with acute pancreatitis. The pancreas may be contributing to increased FGF21 levels following injury and FGF21 may play a role in the recovery process.

## Introduction

FGF21, a member of the endocrine FGF subfamily, is expressed in multiple organs and has several metabolic actions involving both glucose homeostasis and fatty acid oxidation [1–4]. In humans, serum levels are increased in obesity and correlate with fat accumulation in the liver [5–8]. While FGF21 expression is highest in the pancreas[9], few studies have addressed the possible role of FGF21 in this tissue. In mice, increased pancreatic FGF21 is seen with cerulein-induced pancreatic injury, with more severe injury observed in mice lacking FGF21 [10, 11]. To assess the possibility that FGF21 is released from the pancreas during acute injury in humans, we measured circulating FGF21 in subjects with acute pancreatitis over the course of their disease. We observed that acute pancreatitis subjects had significantly increased peak circulating FGF21 values compared to healthy controls. Further, peak FGF21 levels were observed four to six days after lipase levels peaked. The timing of this elevation suggests that FGF21 may play a role in or be a marker of disease recovery.

## Materials and Methods

Medical records of patients admitted to the Beth Israel Deaconess Medical Center (BIDMC) in Boston, MA between May and September 2012 were prospectively reviewed using electronically available team census lists. Twenty five subjects with acute pancreatitis were selected for analysis. A diagnosis of acute pancreatitis was based on the presence of two out of the following three criteria: abdominal pain, lipase greater than three times the upper limit of normal, or radiographic findings of pancreatitis on Computed Tomography (CT) scan. Exclusion criteria included pre-existing malignancy and diabetic ketoacidosis. Hospital course data and demographic information were abstracted from the electronic medical record. Twenty healthy subjects without pancreatitis were used as controls. Each control subject had three baseline serum samples drawn over the course of four weeks for a study assessing the metabolic response to fructose [12] but the variation of baseline levels was not previously analyzed.

Pancreatitis severity was graded according to the Atlanta classification system [13]. Subjects were classified as having moderately severe pancreatitis if they developed local complications or organ failure lasting less than 48 hours, while those with severe pancreatitis developed persistent organ failure; in the absence of these complications, subjects were classified as having mild pancreatitis. Local complications were defined as one of the following:

1. Acute peripancreatic fluid collection causing significant clinical symptoms
2. Pancreatic pseudocyst formation
3. Necrotizing pancreatitis
4. Gastric outlet dysfunction
5. Splenic or portal vein thrombosis

Organ failure was defined by one of the following:

1. Ratio of PaO_2_ to FiO_2_ > 200
2. Serum creatinine > 1.9 mg/dL after fluid resuscitation
3. SBP < 90 mm Hg not responsive to intravenous fluid administration

Discarded serum collected during hospitalization for clinical indications was obtained from the pancreatitis subjects for FGF21 measurements. When multiple blood samples were drawn on a subject during one hospital day, the earliest sample drawn was accessioned and analyzed. Serum samples stored at 4°C in the clinical laboratory were accessioned 4–6 days after being drawn and were then stored at -80°C until analyzed for FGF21 levels. All samples were anonymized prior to retrieval. Control samples for assaying active and total FGF21 were collected as part of a previous metabolic study and had been stored at -80°C. Approval for this research protocol was obtained from the Institutional Review Board (IRB) of BIDMC. A waiver of informed consent was granted by the IRB to collect discarded and anonymized serum samples from patients with acute pancreatitis. Written informed consent approved by the IRB was obtained prior to prospectively collecting the control samples.

The onset of acute pancreatitis was defined as the subject’s reported time of onset of abdominal pain rather than admission to the hospital. “Day 1” levels, for example, refer to serum drawn within 24 hours of abdominal pain. “Day 0” levels, when available, refer to those samples obtained within 24 hours prior to the onset of pancreatitis.

Concentrations of total human FGF21 were measured through a commercially available enzyme-linked immunosorbent assay (ELISA) kit (R&D systems, Minneapolis, MN). Active FGF21 was measured using an ELISA kit from Eagle BioSciences, Nashua, NH. Statistical analysis was performed using GraphPad Prism 7.00 software (San Diego, CA). Student’s t-test and Pearson’s correlation coefficient were used for comparisons between two variables. One-way ANOVA was used to compare active FGF21 levels between control subjects and acute pancreatitis subjects. Two-way ANOVA was used to compare the total versus active peptide from the serum samples that had the highest and lowest FGF21 levels. Statistical significance was defined as a P-value < 0.05.

## Results

We enrolled twenty-five subjects admitted to BIDMC with acute pancreatitis between May and September 2012. S1 Table summarizes demographic information and Table 1 summarizes disease course and laboratory values. All but three subjects presented to the hospital and had blood drawn within 72 hours of the onset of symptoms. Nine subjects had pre-existing gallstones and seven had a history of alcohol abuse. Seventeen subjects had mild pancreatitis, five had moderately severe pancreatitis, and three had severe pancreatitis as defined by the Atlanta Classification criteria [13].

**Table 1:** Pancreatitis Characteristics and Outcomes.

	Number (%) or Value (±SEM)
**Pancreatitis History**	
Prior episode of pancreatitis	6 (24%)
Chronic pancreatitis	3 (12%)
**Time from onset of symptoms to first serum sample (hours)**	
<24	13 (52%)
<48	18 (72%)
<72	22 (88%)
<96	24 (96%)
<120	25 (100%)
**Etiology**	
Gallstones	9 (36%)
Alcohol	7 (28%)
Post-procedural	3 (12%)
Acute on Chronic	3 (12%)
Other	4 (16%)
**Laboratory Values**	
Peak AST (IU/L)	160 ± 46
Peak ALT (IU/L)	189 ± 45
Peak Lipase (IU/L)	3279 ± 709
**Severity of Pancreatitis**	
Mild	17 (68%)
Moderately Severe	5 (20%)
Severe	3 (12%)
**Local Pancreatic Complications**	
Pseudocyst	3 (12%)
Necrotizing Pancreatitis	3 (12%)
Symptomatic Peripancreatic Fluid Collection	4 (16%)
**Renal Failure (Creatinine > 1.9 mg/dL)**	2 (8%)
Transient (lasting < 48 hours)	1 (4%)
Persistent (lasting > 48 hours)	1 (4%)
**Cardiovascular Failure (SBP < 90 mm Hg and not fluid responsive)**	1 (4%)
Transient	0 (0%)
Persistent	1 (4%)
**Respiratory Failure (PaO**_**2**_ **to FiO**_**2**_ **ratio < 200)**	3 (12%)
Transient	0 (0%)
Persistent	3 (12%)
**Intensive Care Unit Admission**	7 (28%)
**Mortality**	1 (4%)

SEM, standard error of mean; AST, aspartate aminotransferase; ALT, alanine aminotransferase; SBP, systolic blood pressure; PaO_2_, partial pressure of arterial oxygen; FiO_2_, fraction of inspired oxygen.

**Fig 1:**
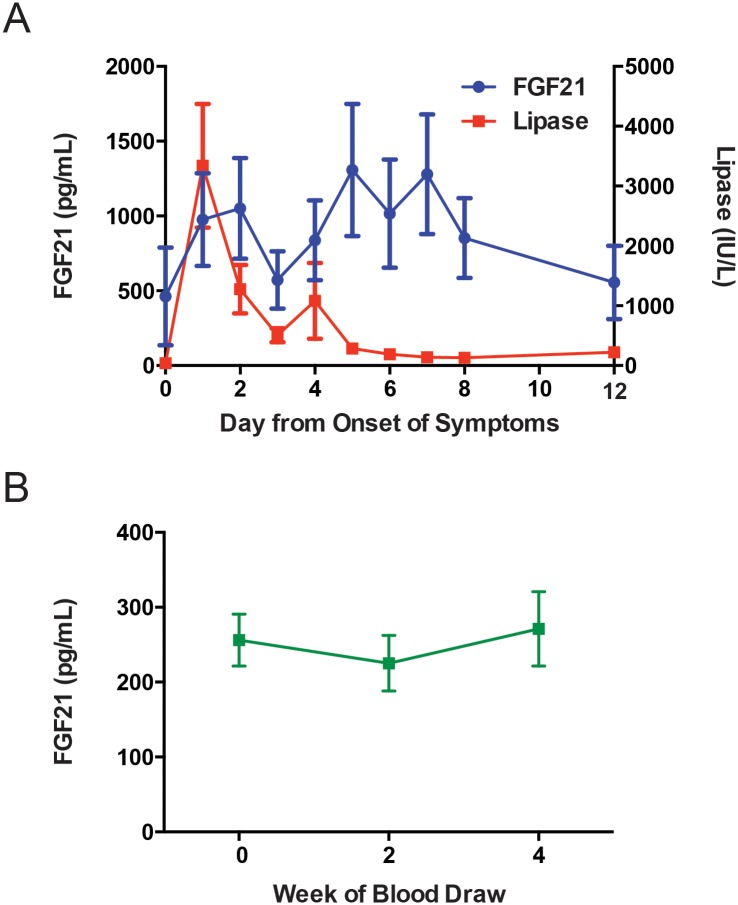
FGF21 and lipase levels in acute pancreatitis subjects and FGF21 levels in control subjects. Panel A shows mean serum FGF21 and lipase levels in acute pancreatitis subjects from the day of onset of symptoms, while panel B shows mean FGF21 levels in healthy control subjects drawn every two weeks over the course of four weeks. Data shown as the mean ± SEM.

FGF21 serum levels peaked between five and seven days from the onset of symptoms (maximum level on day 5) or four to six days after the lipase peak (Fig 1A). To evaluate possible natural variation in serum FGF21 levels, we measured FGF21 in twenty individuals who had served as healthy volunteers in a previous study [12]. These samples were obtained on three independent occasions over the course of four weeks. We controlled for the known variation of FGF21 with BMI by selecting control subjects within the same range of BMI as the pancreatitis subjects (28.6 (range 21–35.5) vs. 29.3 (range 19.1–46) for acute pancreatitis subjects, P = 0.73). In contrast to the average FGF21 levels in subjects with acute pancreatitis, average FGF21 levels in this cohort of control subjects remained quite stable (range 225.3 ± 38.1 to 271.2 ± 52.3, P = ns) (Fig 1B).

**Fig 2:**
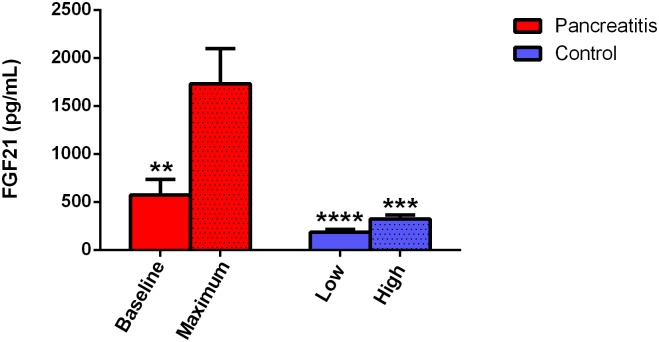
Maximum FGF21 levels in acute pancreatitis are significantly greater than baseline levels and control levels. The baseline FGF21 level for acute pancreatitis subjects is the lower value of either the first or last measured FGF21 level during a hospital course. Data shown as mean ± SEM. N = 25 for acute pancreatitis, N = 20 for control subjects. ** P < 0.01 compared to maximum levels; *** P < 0.001 compared to maximum levels; **** P < 0.0001 compared to maximum levels.

As subjects did not present at a uniform time during the course of acute pancreatitis, we arbitrarily designated a subject’s “baseline” FGF21 level as the lower value of either the first or last measured FGF21 level during hospitalization. Maximum FGF21 levels for the acute pancreatitis subjects were significantly greater than baseline levels (Fig 2, 1733 vs. 638 pg/mL, P = 0.003). We also compared these values to the highest and lowest values of FGF21 obtained for control subjects. Maximum FGF21 in acute pancreatitis subjects was significantly greater than the highest FGF21 values in controls (Fig 2, 1733 vs. 322 pg/mL, P = 0.0002). There was no statistically significant difference observed between the lowest value for control groups and baseline levels in acute pancreatitis patients (P = 0.50). Likewise, there was no significant difference between the highest and lowest values of FGF21 in control subjects (Fig 2, 322 vs. 184 pg/mL, P = 0.98). Fold changes for acute pancreatitis subjects were significantly greater than fold changes for controls (4.7 vs. 2.0, P = 0.01). These findings suggest that the rise in FGF21 seen in acute pancreatitis subjects is distinct from the natural fluctuations observed in healthy subjects.

Active FGF21 levels were analyzed in those samples containing the highest and lowest total FGF21 levels for each acute pancreatitis subject. Six subjects were excluded from this analysis either because FGF21 levels were below the lower limit of detection for the assay or because active levels were calculated as paradoxically higher than total FGF21 levels. We found that the range of the percent of active to total FGF21 was similar for the highest and lowest FGF21 levels for each subject (3.1–89.9% for high, 11.1–91.7% for low) and was comparable to the range of values seen for healthy controls (19.7–97.3%, N = 4). As well, there was no statistically significant difference in percent active FGF21 between these three groups (42.5 ± 5.4% for high, 44.4 ± 5.0% for low, 56.0 ± 16.0% for controls, P = 0.58). In any given individual, there was a high correlation between the percent active FGF21 at the point when they had their lowest and highest levels (S1 Fig, R^2^ = 0.37, P = 0.005). The stability of this ratio indicates that the increases observed during the course of disease do not reflect accumulation of inactive FGF21.

Maximum FGF21 levels did not correlate with pancreatitis severity; however, the fold increase was greater in severe compared to mild but not moderately severe pancreatitis (4.4 ± 1.0 for mild, 5.5 ± 1.6 for moderately severe, 18.2 ± 11.8 for severe, P = 0.01 mild vs. severe). Higher fold increases were also noted in those with pancreatic necrosis versus those without (18.3 ± 11.7 vs. 4.6 ± 0.8, P = 0.004).

Maximum but not baseline FGF21 levels were higher in subjects with alcohol-induced pancreatitis compared to those with pancreatitis of a different etiology (S2 Fig, 3077 ± 897 vs. 1210 ± 310 pg/mL, P = 0.02). Absolute changes in FGF21 levels were significantly greater in those admitted to the intensive care unit (2439 ± 907 vs. 665 ± 155 pg/mL, P = 0.007).

We next analyzed the relationship between FGF21 levels and clinical outcomes. There was no association with improvements in clinical status such as transition from parenteral to oral pain medication or from the ICU to the hospital floor. However, in those twenty one subjects discharged home tolerating oral alimentation, day of maximum FGF21 levels correlated with the day of successful return to oral intake (R^2^ = 0.21, P = 0.04), and both events occurred within two days of each other in all but one subject. There was no statistically significant difference in FGF21 levels before or after the initiation of oral intake, suggesting that return to the fed state from fasting did not cause FGF21 levels to peak (S3 Fig, 1248 ± 523 before oral intake vs. 1229 ± 387 pg/mL, P = 0.96).

In otherwise healthy populations, FGF21 increases with increasing body mass index (BMI) [5], however we found a trend towards an inverse correlation between BMI and maximum FGF21 levels (S4 Fig, R^2^ = 0.12, P = 0.09) as well as BMI and baseline FGF21 levels (R^2^ = 0.15, P = 0.05).

The amplitude of maximum FGF21 levels did not correlate with that of peak lipase levels. Likewise, there were no associations between FGF21 and potentially confounding factors, such as those noted in S1 Table (e.g. age, sex, diabetes, hyperlipidemia, hypertension, medications, or serum glucose levels). In addition, there were no associations between FGF21 levels and pseudocyst formation, peripancreatic fluid collection, or prior history of acute or chronic pancreatitis. Liver disease had no association with FGF21 levels in the context of acute pancreatitis, as no relationship was observed between FGF21 levels and non-alcoholic fatty liver disease, AST, or ALT elevation in these subjects.

## Discussion

Our study demonstrates that, in subjects with acute pancreatitis, circulating levels of FGF21 rise substantially and peak around the time of return to oral intake. This timing suggests that increased FGF21 reflects the recovery phase of the acute disease. Maximum FGF21 was significantly greater than baseline FGF21 in acute pancreatitis subjects and significantly greater than levels seen for healthy controls. Relative changes in FGF21 may be associated with pancreatitis severity, and subjects with alcohol-induced pancreatitis tended to have higher maximum FGF21 levels. Previous reports in otherwise healthy humans demonstrated a positive correlation between serum FGF21 levels and BMI, but this correlation is inverted in our subjects [5–8].

Our findings are consistent with reports showing that pancreatic FGF21 expression increases in mice with cerulein-induced pancreatitis [10, 11]. To our knowledge, only one other study has analyzed FGF21 levels in human acute pancreatitis [14]. In contrast to our findings, FGF21 levels in acute pancreatitis subjects in this report declined between days one and four of hospitalization [14]. This was also accompanied by a reduction in IL-6 levels between hospital days one and four, suggesting that subjects in this European study presented later in the course of their disease than the American counterparts in our study. Nonetheless, the finding of a rise of FGF21 in acute pancreatitis is consistent.

This is the first report comparing total to active FGF21 levels in humans. Our findings in acute pancreatitis suggest that the percentage of total FGF21 that is active in an individual remains stable throughout the course of disease and that increases in FGF21 are not merely due to accumulation of inactive peptide; thus the observed increase results from both increased active and inactive forms.

There is a range of FGF21 serum levels in healthy lean humans. Baseline FGF21 levels in our acute pancreatitis subjects are comparable to those reported in other studies [5–8] [15–17]. The increase in FGF21 observed during the disease course in the acute pancreatitis subjects is significantly greater than the baseline fluctuations in our control subjects; this finding suggests that absolute and fold FGF21 changes in acute pancreatitis reflect disease course and not fluctuation over time. It is also possible that the actual fold-increase in our subjects is higher, as the lowest level recorded during the course of the hospital stay may not accurately reflect a subject’s true baseline FGF21 level when healthy.

Our study has some limitations. First, samples from pancreatitis patients were obtained from stored blood taken for clinical indications rather than at regularly timed intervals. Although these specimens were often drawn in the early morning, they were not necessarily drawn daily, leading to gaps in the data for some subjects. In addition, subjects did not necessarily present at the initial onset of symptoms. Using the electronic medical records, however, we were able to ascertain the time point in their disease that subjects had their initial blood draw. Finally, most subjects did not have true baseline FGF21 levels available as samples were collected during the hospital stay and no values were available once subjects were back in their usual state of health.

We have shown that serum FGF21 levels are increased in human pancreatitis, which is consistent with prior data in mice and humans. Although the exact reason for the increase is unclear, this finding may be secondary to pancreatitis-induced inflammation, as FGF21 has been shown to play a role in other inflammatory conditions and disease states such as myocardial infarction [18–21]. To date, it is difficult to identify the tissue of origin of circulating FGF21 in humans for most disease states. One study, limited to healthy exercising males, reports that increased circulating FGF21 during exercise derives from the liver [22]. In studies of human mitochondrial myopathies, muscle was identified as the source of FGF21 by examining expression of FGF21 in muscle biopsy specimens [23]. In the context of disease, however, the source of FGF21 cannot be readily identified, as making this determination would require direct sampling of the arterial and venous supply of various potential organs which is beyond the capability of most human studies. We speculate, however, that the tissue of origin is the acinar pancreas, which is supported by previous research showing that FGF21 in the mouse pancreas is most highly expressed in the acinar compartment [24]. Typically, circulating FGF21 derives from the liver [25] and is associated with BMI and the presence of fatty liver. It therefore seems unlikely that the liver contributes to the spike in FGF21 in this cohort both because of the inverse correlation with BMI observed and because there was no association with the presence of fatty liver and hepatic injury. We speculate that the rise in serum FGF21 reflects a potential role in disease recovery, but more studies are needed to elucidate the precise role of FGF21 in acute pancreatitis.

## Supporting Information

S1 Fig Ratio of active to total FGF21 is directly correlated for low and high values in subjects with acute pancreatitis. Ratio of active to total FGF21 for the lowest total FGF21 level for a subject is plotted against the ratio for the highest total FGF21 level for the same subject. N = 19.
(TIF)

S2 Fig Maximum FGF21 levels are higher in alcohol-induced pancreatitis than in pancreatitis of other etiology. Error bars are standard error of mean. * P < 0.05
(TIF)

S3 Fig Initiation of oral alimentation does not increase FGF21 levels. FGF21 levels for individual subjects within 24 hours before and after the initiation of oral intake. Serum samples were available for analysis in seventeen subjects.
(TIF)

S4 Fig Maximum FGF21 levels and BMI trend towards a negative correlation in subjects with acute pancreatitis. (TIF)

S1 Table Subject Demographics and Health Characteristics for Acute Pancreatitis Subjects. Medications listed are those that were being taken on an outpatient basis. SEM, standard error of the mean; BMI, body mass index.
(DOCX)
