# Supplementary figures and images for: Elevated Serum Fibroblast Growth Factor 21 in Humans with Acute Pancreatitis
Source: PLoS One. 2016 Nov 10;11(11):e0164351. doi: 10.1371/journal.pone.0164351 (PMC5104316; doi:10.1371/journal.pone.0164351)

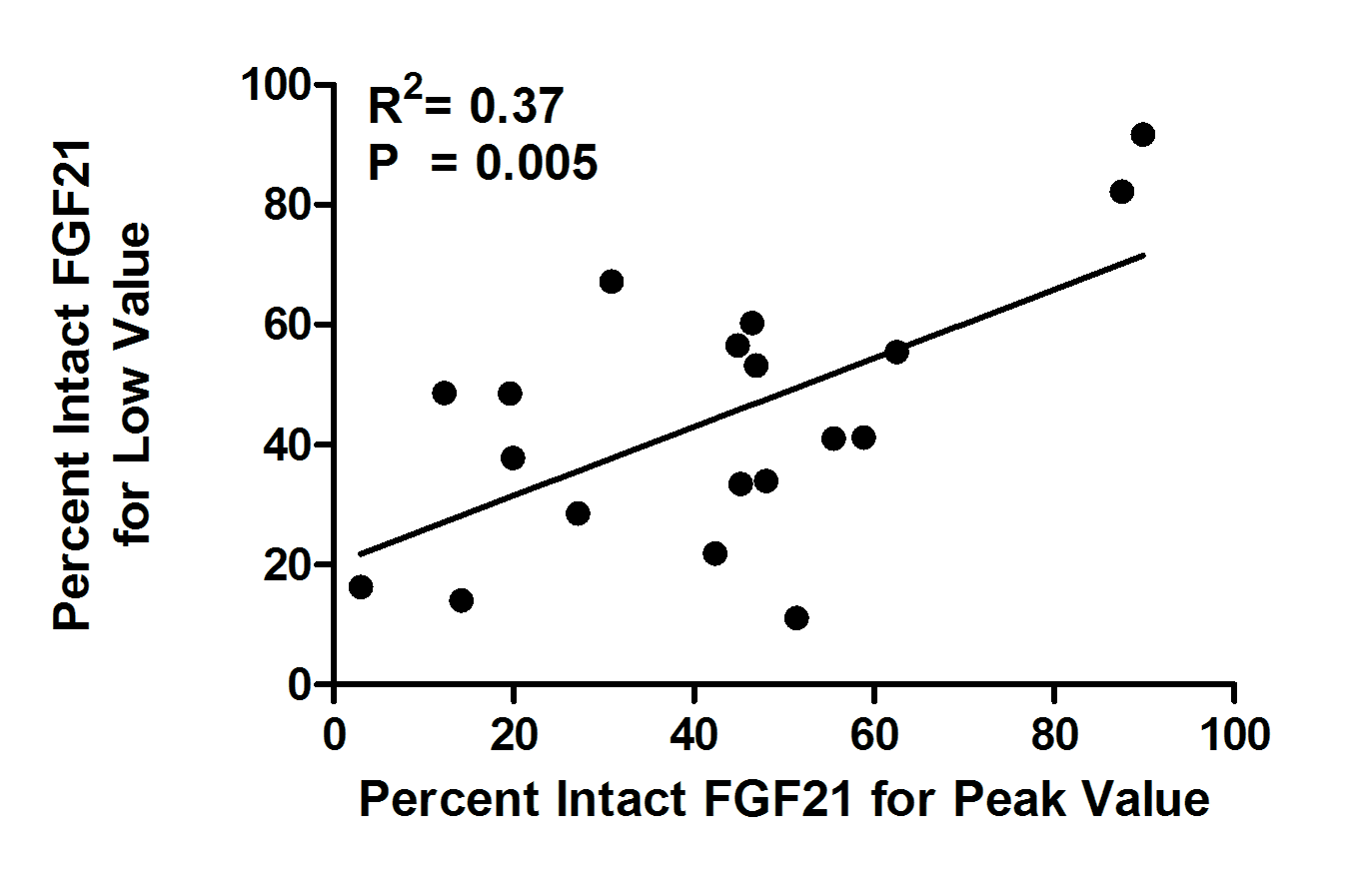

Supplement: S1 Fig — Ratio of active to total FGF21 for the lowest total FGF21 level for a subject is plotted against the ratio for the highest total FGF21 level for the same subject. N = 19. (TIF) [file pone.0164351.s001.tif]

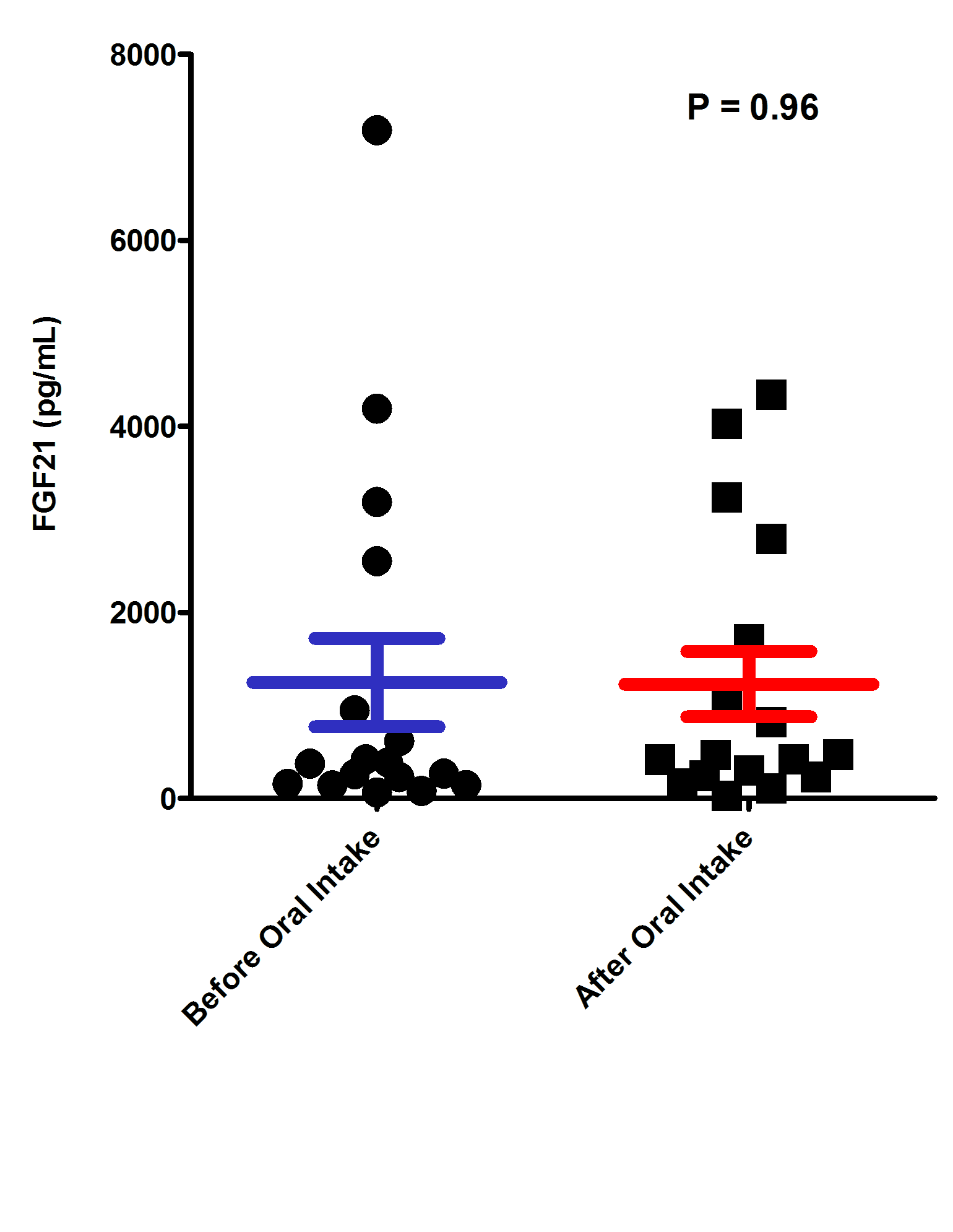

Supplement: S3 Fig — FGF21 levels for individual subjects within 24 hours before and after the initiation of oral intake. Serum samples were available for analysis in seventeen subjects. (TIF) [file pone.0164351.s003.tif]
