# Supplementary material for: Elevated Serum Fibroblast Growth Factor 21 in Humans with Acute Pancreatitis
Source: PLoS One. 2016 Nov 10;11(11):e0164351. doi: 10.1371/journal.pone.0164351 (PMC5104316; doi:10.1371/journal.pone.0164351)
Supplement: S1 Table — Medications listed are those that were being taken on an outpatient basis. SEM, standard error of the mean; BMI, body mass index. (DOCX) [file pone.0164351.s005.docx]

| Subject Characteristic | Number (± SEM) |
| --- | --- |
| Age (years) | 51.8 ± 3.4 |
| Sex |  |
| Male | 12 |
| Female | 13 |
| Length of Hospital course (Days) | 12.0 ± 2.9 |
| BMI | 29.6 ± 1.5 |
| Preexisting Comorbidities |  |
| Obesity | 8 |
| Diabetes | 5 |
| Hyperlipidemia | 11 |
| Hypertension | 12 |
| Fatty Liver | 10 |
| Medications |  |
| Statins | 6 |
| Beta-blockers | 8 |
| Fibrates | 2 |
| Metformin | 1 |
| Insulin | 3 |
| Sulfonylureas | 6 |

**S1 Table**. **Subject Demographics and Health Characteristics for acute pancreatitis subjects.**  Medications listed are those that were being taken on an outpatient basis. SEM, standard error of the mean; BMI, body mass index.
